# Combination of anti-L1 cell adhesion molecule antibody and gemcitabine or cisplatin improves the therapeutic response of intrahepatic cholangiocarcinoma

**DOI:** 10.1371/journal.pone.0170078

**Published:** 2017-02-06

**Authors:** Seulki Cho, Tae Sup Lee, In Ho Song, A-Ram Kim, Yoon-Jin Lee, Haejung Kim, Haein Hwang, Mun Sik Jeong, Seung Goo Kang, Hyo Jeong Hong

**Affiliations:** 1 Department of Functional Genomics, University of Science & Technology, Daejeon, Republic of Korea; 2 Institute of Bioscience and Biotechnology, Kangwon National University, Chuncheon, Republic of Korea; 3 Division of RI Convergence Research, Korea Institute of Radiological and Medical Sciences, Seoul, Republic of Korea; 4 Division of Basic Radiation Bioscience, Korea Institute of Radiological & Medical Sciences, Seoul, Republic of Korea; 5 Department of Biology, Kangwon National University, Chuncheon, Republic of Korea; 6 Department of Systems Immunology, Kangwon National University, Chuncheon, Republic of Korea; University of South Alabama Mitchell Cancer Institute, UNITED STATES

## Abstract

Cholangiocarcinoma has a poor prognosis and is refractory to conventional chemotherapy and radiation therapy. Improving survival of patients with advanced cholangiocarcinoma urgently requires the development of new effective targeted therapies in combination with chemotherapy. We previously developed a human monoclonal antibody (mAb) Ab417 that binds to both the human and mouse L1 cell adhesion molecule (L1CAM) with high affinities. In the present study, we observed that Ab417 exhibited tumor targeting ability in biodistribution studies and dose-dependent tumor growth inhibition in an intrahepatic cholangiocarcinoma (Choi-CK) xenograft mouse model. Regarding the mechanism of action, Ab417 was internalized into the tumor cells and thereby down-regulated membrane L1CAM, and inhibited tumor growth by reducing tumor cell proliferation *in vivo*. Gemcitabine inhibited the tumor growth in a dose-dependent manner in the Choi-CK xenograft model. However, cisplatin inhibited the tumor growth moderately and not in a dose-dependent way, suggesting that the tumors may have developed resistance to apoptosis induced by cisplatin. Combined treatment with Ab417 and gemcitabine or cisplatin exerted enhanced tumor growth inhibition compared to treatment with antibody or drug alone. The results suggest that Ab417 in combination with chemotherapy may have potential as a new therapeutic regimen for cholangiocarcinoma. Our study is the first to show an enhanced therapeutic effect of a therapeutic antibody targeting L1CAM in combination with chemotherapy in cholangiocarcinoma models.

## Introduction

Cholangiocarcinoma is an aggressive malignancy arising from the ductal epithelium of the biliary tract. It can be classified anatomically as intrahepatic cholangiocarcinoma (ICC) or extrahepatic cholangiocarcinoma (ECC) [1, 2]. Cholangiocarcinoma has a poor prognosis; it is notoriously difficult to diagnose because of its late clinical presentation and is refractory to conventional chemotherapy and radiation therapy [1]. Thus, the overall 5-year survival rate of cholangiocarcinoma patients is 5–10%, and even in cases involving potentially curative surgery, the reported 5-year survival rates are 25–30% [3]. Therefore, there is a clear need for new and effective therapeutic regimens that improve survival of cholangiocarcinoma patients.

Gemcitabine, a pyrimidine analog that inhibits DNA synthesis, has been extensively investigated as a chemotherapeutic drug for cholangiocarcinoma. In a phase II study, gemcitabine monotherapy yielded moderate efficacy with manageable toxicity in patients with advanced or metastatic cholangiocarcinoma [4]. In randomized phase II and III trials, gemcitabine plus cisplatin was associated with a significant survival advantage compared with gemcitabine alone in patients with locally advanced or metastatic biliary tract cancer, suggesting that gemcitabine plus cisplatin (or oxaliplatin) is the standard regimen for advanced biliary tract cancer [5–7]. However, response to the combination chemotherapy in cholangiocarcinoma patients is limited, and the 5-year survival remains low [8]. The epidermal growth factor receptor (EGFR) signaling pathway regulates biliary epithelial cell growth, and EGFR is overexpressed in biliary tract cancers [9, 10]. Recently, addition of the anti-EGFR antibody cetuximab to gemcitabine plus oxaliplatin did not enhance the efficacy of chemotherapy in patients with advanced biliary tract cancers in a randomized phase II trial [8, 11]. Also, a recently published randomized phase III trial of erlotinib, an EGFR tyrosine kinase inhibitor, in combination with gemcitabine and oxaliplatin, when compared with the chemotherapy doublet, showed no obvious improvement in clinical outcomes [12]. Therefore, new targeted therapies in combination with chemotherapy that offer improved patient survival are highly needed.

L1 cell adhesion molecule (L1CAM, CD171) is a 200–220-kDa transmembrane glycoprotein composed of 6 immunoglobulin-like domains, 5 fibronectin-type III domains, a transmembrane stretch, and a short cytoplasmic tail [13]. L1CAM was originally identified as a neural cell adhesion molecule in the central nervous system that plays an important role in initiating cerebellar cell migration and neurite outgrowth [14]. L1CAM expression is also found in other cell types such as lymphoid and myelomonocytic cells, kidney tubule epithelial cells, and intestinal crypt cells [15–18]. In addition, L1CAM expression has been identified in a variety of tumor types and correlates with poor prognosis and metastasis [19]. L1CAM functions mostly in proliferation, migration, invasion, and survival through L1CAM homophilic interaction or heterophilic interactions with other cell adhesion molecules, integrins, or growth factor receptor, while the cellular properties are not homogeneous among different types of cancers [20]. Recently, we found that L1CAM is aberrantly expressed in biliary tract cancers such as ICC, ECC, and gall bladder carcinoma (GBC), and its high expression correlates with poor prognosis and metastasis [19–21]. Also, L1CAM acts as an independent poor prognostic factor predicting patient survival in ECC and GBC [21–23]. In addition, increased expression of L1CAM enhances proliferation, migration, invasion, and/or cisplatin resistance of ICC and GBC cells and tumor growth *in vivo*, while down-regulation of L1CAM reduces them [21, 24, 25]. These results suggest that L1CAM may be a promising new target for the treatment of biliary tract cancers.

Monoclonal antibodies (mAbs) have proved to be effective therapeutic agents for the treatment of cancers because of their antigen-binding specificities and ability to trigger the immune system [26]. We previously developed a fully human anti-L1CAM mAb (Ab417, IgG1) that cross-reacts with mouse L1CAM, and validated that Ab417 inhibits tumor growth in a Choi-CK xenograft nude mouse model [27]. In the present study, to gain insight into whether a combination of Ab417 and chemotherapy offers a new effective targeted therapy for cholangiocarcinoma, we investigated the mode of action of Ab417 and evaluated the therapeutic efficacies of Ab417, chemodrugs (gemcitabine or cisplatin), and combined treatment with Ab417 and chemodrug (gemcitabine or cisplatin).

## Materials and methods

### Cell lines and cell culture

The human ICC cell line Choi-CK [28] was kindly provided by Dr. Dae-Gon Kim at Chunbuk National University. Choi-CK and SCK-L1 [21] cells were cultured in DMEM (Welgene, Republic of Korea) supplemented with 10% fetal bovine serum (FBS) (Atlas Biologics, USA). The CHO-DG44 stable cell line #9–20 [27] expressing Ab417 antibody was cultured in MEM-α (Welgene) supplemented with 5% (v/v) dialyzed FBS (Thermo Fisher Scientific) and changed to serum-free medium (SFM4CHO, GE Lifesciences, USA) for the production of antibody. All cell lines were cultured at 5% CO_2_, 37°C in a humidified incubator.

### Production of Ab417

Ab417 was produced from the stable CHO-DG44 cell line (#9–20). The culture supernatant was centrifuged and filtered using a bottle top filter (0.22 μm PES, Sartorius) and then subjected to affinity chromatography on a protein A-agarose (GenScript, USA) for purification. Protein A–bound antibodies were eluted using 0.1 M sodium citrate containing 150 mM sodium chloride (pH 3.6). Eluted antibody was stored after buffer change to 25 mM sodium citrate containing 150 mM sodium chloride and 0.007% Tween-20 (pH 6.4). The protein concentration was determined with a NanoDrop 2000 UV-Vis Spectrophotometer (Thermo Fisher Scientific).

### *In vivo* anti-tumor efficacy studies

Nude mice (BALB/c Slc-*nu*, 5 weeks old) were obtained from Japan SLC, Inc. (Japan) and housed under specific pathogen-free conditions for 7 days in accordance with the guidelines of the Animal Care Committee at Biotoxtech (Republic of Korea). The animal studies to evaluate anti-tumor efficacy of Ab417, drug (gemcitabine or cisplatin), or combination of Ab417 and drug (gemcitabine or cisplatin) were approved prior to beginning studies from the committee (B13698, B13933, or B13934, respectively). Prior to tumor cell injection, mice were anesthetized briefly with inhalation of isoflurane (0.8–1.1% for induction and maintenance) in a standard anesthesia chamber. Choi-CK cells (1 × 10^6^) were inoculated subcutaneously into the right flank of each mouse. The tumors formed were sectioned and the resulting Choi-CK tumor tissue (3 × 3 × 3 mm^3^) was transplanted subcutaneously into the backs of new nude mice. When tumor volume reached approximately 100 mm^3^, mice were randomly divided into groups, and antibody or drug (gemcitabine or cisplatin) was *i*.*v*. or *i*.*p*., respectively, injected twice a week (*n* = 8 per group). Gemcitabine (gemcitabine hydrochloride, Sigma Aldrich) and cisplatin *(cis*-Diammineplatinum (II) dichloride, Sigma Aldrich) were dissolved at appropriate concentrations by injecting sodium chloride (Choongwae, Republic of Korea). Extra animals not selected for the study were euthanized by CO_2_ exposure.

Tumor growth was monitored by measuring the length and width of the tumor with a caliper and calculating tumor volume on the basis of the following formula: TV (mm^3^) = L (mm) × W^2^ (mm^2^) × 1/2, where L is length and W is width. Body weight of the animals was also measured. At the end of the experiments, animals were anesthetized by using isoflurane, then tumor tissues were taken out and weighed. Tumor growth inhibition rate (IR) was calculated as follows: IR (%) = (1—T/C) × 100, where T is the mean tumor weight of the test substance group and C is mean tumor volume or weight of the negative control group.

To elucidate the mechanism of action, nude mice (BALB/c-nude, 6 weeks old) were obtained from NARA Biotech (Republic of Korea) and housed under specific pathogen-free conditions for 7 days in accordance with the Kangwon University IACUC (KW-160429-1). Choi-CK cells (1 × 10^6^) were inoculated subcutaneously into the right flank of nude mouse (*n* = 3). When tumor volume reached approximately 110 mm^3^, mice were randomly divided into 2 groups, and antibody or PBS was *i*.*p*. injected thrice a week. At 10 days postinjection, tumors were taken out and subjected to immunohistochemistry for detecting Ki-67 and immunofluorescent staining for detecting L1CAM. After experiments, all animals were euthanized by CO_2_ exposure.

### Immunohistochemical analysis

Mice were euthanized and tissues were harvested and fixed in formalin for the preparation of paraffin sections. Paraffin-embedded tissue sections were deparaffinized in xylene, 95%, 90%, 70%, and 50% ethanol, followed by washing with PBS. De-paraffinized slides were boiled in 0.1 mol/L citrate buffer (pH 6.0) for 30 min and next incubated with 0.3% (v/v) hydrogen peroxide in methanol for 15 min. Sections were blocked in normal horse serum at room temperature (RT) for 30 min and immunostained overnight at 4°C with primary antibodies against Ki-67 (C-term) (1:200, Acris, Germany). The target proteins were visualized using ABC and DAB kits (Vector Laboratories, USA) and counterstained with hematoxylin.

For immunofluorescence staining, slides were immunostained overnight at 4°C with anti-NCAM-L1 (N-14) mAb (1:50, Santa Cruz Biotechnology, USA), followed by incubation for 1 h at RT with donkey anti-goat Alexa 488-conjugated secondary antibodies (1:200, Thermo Fisher Scientific). Cell nuclei were labeled with DAPI (5 μM, Sigma Aldrich). Images were captured using a Zeiss confocal microscope. Image J software (version 1.45) was used for quantitative evaluation of the staining.

### Antibody internalization assay

Internalization of Ab417 was measured by confocal laser scanning microscopy. Warm culture medium containing Ab417 (10 μg/mL) was added to L1CAM-overexpressing SCK-L1 cells plated on a slide glass. After incubation at 37°C for 0.5 or 4 h, the cells were placed on ice, rinsed with 4°C PBS, and fixed with 4% formaldehyde (Sigma Aldrich). As a zero-time control, Ab417 was added to formaldehyde-fixed cells. Cell surface antibody was detected with a rabbit anti-human IgG(H+L) Cross Adsorbed DyLight 594 polyclonal antibody (1:100, v/v, Thermo Fisher Scientific) for 5 min at room temperature. The cell surface secondary antibody was post-fixed for 5 min using formaldehyde. After washing, cells were blocked and permeabilized with 10% goat serum and 0.1% Triton X-100 in PBS for 30 min at room temperature. To label internalized L1CAM, cells were incubated with anti-human IgG (Fc- specific)-FITC (1:500, v/v) in PBS containing 5% goat serum and 0.02% Triton X-100 for 30 min at room temperature. The slides were washed three times with PBS and mounted using the SlowFade® Antifade kit (Thermo Fisher Scientific). Fluorescence signals were detected with an Olympus LX70 FV300 05-LGP-193 (OLYMPUS, Japan).

### Analysis of membrane L1CAM level in Ab417-treated cells

Choi-CK cells were incubated with Ab417 (10 μg/mL) for the indicated time, and membrane protein fractions were obtained with MEM-PER^TM^ Plus Membrane Protein Extraction Kit (Thermo Fisher Scientific). The obtained protein samples were quantified by a BCA protein quantification assay and subjected to SDS-PAGE. After transfer to nitrocellulose membrane and blocking with Tris-buffered saline containing 0.1% Tween-20 and 5% skim milk (BD Biosciences), L1CAM was detected using a mouse mAb binding to human L1CAM [21] and goat anti-mouse IgG-HRP conjugate (1:8,000, v/v, Thermo Fisher Scientific). Pan-cadherin was used as a loading control with the Pan-cadherin rabbit mAb (1: 2,500, v/v, Cell Signaling Technology, USA) and anti-rabbit IgG-HRP conjugate (1:5,000, v/v, Cell Signaling Technology). Finally, immunoreactive bands were visualized using a SuperSignal^TM^ West Femto Maximum Sensitive Substrate (Thermo Fisher Scientific).

### Biodistribution study

Ab417 was radiolabeled with ^64^Cu, as described previously [29]. Briefly, NOTA-Ab417 was prepared by incubating 10 mg of Ab417 with 10 equivalents of the bifunctional chelator, 2-S-(4-Isothiocyanatobenzyl)-1,4,7-triazacyclononane-1,4,7-triacetic acid (*p*-SCN-Bn-NOTA, Macrocyclics Inc., USA) in 0.1 M sodium bicarbonate buffer (pH 8.5) at 4°C overnight. NOTA-Ab417 (1 mg in 0.5 mL) was incubated with ^64^Cu solution (37 MBq, KIRAMS, Republic of Korea) in 0.1 M sodium acetate buffer (pH 6.5) for 60 min at room temperature. Radiolabeling yield and radiochemical purity were analyzed by Instant Thin Layer Chromatography-silica gel (ITLC-sg) as a stationary phase and 20 mM citrate buffer with 50 mM EDTA (pH 5.0) as a mobile phase.

For the biodistribution study, 3.7 MBq (100 μg) dose of ^64^Cu-NOTA-Ab417 was injected into each mouse (*n* = 3) bearing a Choi-CK xenograft, and mice were sacrificed at the indicated time points. Blood and normal tissues (heart, liver, lung, spleen, kidney, stomach, intestine, muscle, femur, and brain) were collected, and tumors were also excised. All samples were weighed, and the radioactivity was measured using a NaI crystal well-type gamma counter (Perkin-Elmer, Wizard 1480), applying a decay correction. Counts were compared with those of standards, and the data were expressed as the percentage of injected dose per gram of tissue (%ID/g).

To evaluate the internalization of Ab417, we compared the biodistribution between radioiodine ^125^I-labeled Ab417 and ^64^Cu-Ab417. Ab417 antibody was radiolabeled with ^125^I using Iodogen-coated tube (Thermo Fisher Scientific) by incubating for 30 min. Radiolabeling yield and radiochemical purity were analyzed by ITLC-sg. The ^125^I-Ab417 at the 740 kBq (100 μg) dose was intravenously injected into each mouse (*n* = 3) bearing a Choi-CK xenograft, and mice were sacrificed at each time point for 72 h.

### Cell proliferation assay

Cells (1 × 10^5^) were seeded in a 6-well cell culture plate (SPL, Republic of Korea) in culture medium. After 12 h, when cells attached to the plates, culture media were exchanged to media containing serially diluted gemcitabine or cisplatin (0–3 μg/mL). After 72 h, cells were detached with 0.05% trypsin-EDTA and washed with media. Viable cells were counted using Vi-CELL^TM^ XR (Beckman Coulter, USA), and cell viability was calculated relative to the viable cell number of drug-untreated wells.

### Flow cytometry

To analyze cell cycle arrest by chemodrugs, Choi-CK cells were incubated with cisplatin (Sigma Aldrich) or gemcitabine (Sigma Aldrich) at the indicated concentration, prepared by dissolving in sodium chloride (Choongwae, Republic of Korea). After 48 h, drug-treated or -untreated cells were harvested and prepared in single cell suspension in hypotonic solution containing 0.1% sodium citrate, 0.1% Triton X-100, and 100 μg/mL Ribonuclease A. Shortly before analysis, PI was added to cells, and samples were stored at 4°C. All samples were analyzed using a BD FACSCalibur^TM^ (BD Biosciences, USA), and data were processed with WinMDI ver. 2.9 and Flowing Software ver. 2.5.1.

### Statistical analysis

Data are presented as mean ± s.d., and statistical comparisons between groups were performed using one-way analysis of variance followed by Dunnett’s *t*-test or Steel’s test. A value of *p* < 0.05 was considered significant.

## Results

### Anti-tumor efficacy of Ab417 in a Choi-CK xenograft mouse model

**Fig 1:**
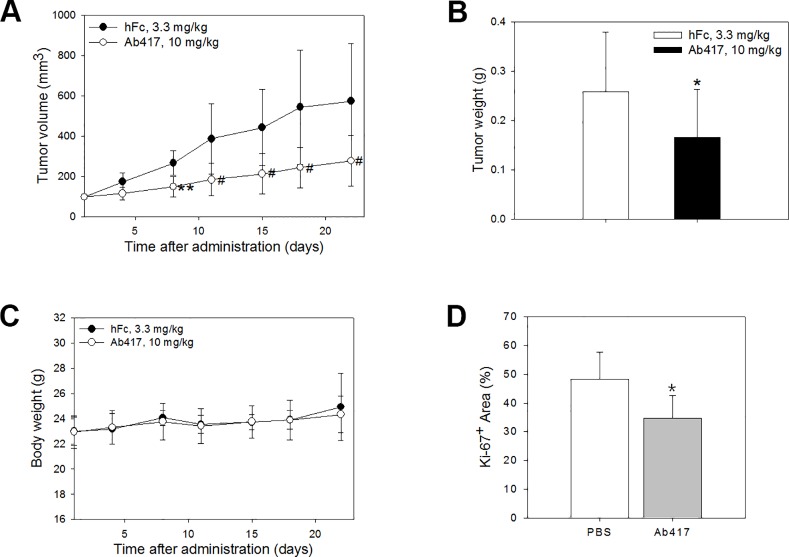
Anti-tumor efficacy of Ab417 in a Choi-CK xenograft model. Ab417 (10 mg/kg) or the isotype control (hFc, 3.3 mg/kg) was *i*.*v*. injected twice a week into nude mice bearing Choi-CK or TFK-1 xenografts (*n* = 8). Tumor volume (A), tumor weight (B), and body weight (C) were represented. Each point indicates the mean ± s.d. *p* < 0.05 (*) and *p* < 0.01 (**), significant difference from the isotype control group by Dunnett’s *t*-test. *p* < 0.05 (^#^), significant difference from the isotype control group by Steel’s test. (D) Tumor sections were stained with an anti-human Ki-67 antibody. Ki-67 index are represented as a percentage of positively-stained area comparing to total area.

In our previous study, we measured the anti-tumor efficacy of Ab417 after the antibody (10 mg/kg) was *i*.*v*. injected three times a week into nude mice (*n* = 8) bearing the Choi-CK xenograft. At 22 days post-injection, Ab417 resulted in 68.6% tumor growth inhibition compared to recombinant human Fc (hFc) as an isotype control, based on mean tumor weight, while it did not affect body weight or induce other adverse effects in the mice [27]. In the present study, to examine if the antibody exhibits anti-tumor efficacy in a dose-dependent manner, Ab417 (10 mg/kg) or hFc (3.3 mg/kg) was *i*.*v*. injected twice a week into the same model (*n* = 8). Ab417 inhibited tumor growth without affecting body weight (Fig 1A–1C). At 22 days post-injection, the mean tumor volume and weight of Ab417-treated groups were 278.3 mm^3^ (*p* < 0.05) and 0.17 g (*p* < 0.05), respectively, while those of the control group were 574.5 mm^3^ and 0.26 g, respectively. Thus, the tumor growth inhibition of an Ab417-treated group was 35.6% compared to hFc, based on mean tumor weight. Taken together, the results indicate that Ab417 exhibits dose-dependent tumor growth inhibition in a Choi-CK xenograft model.

To investigate whether the tumor growth inhibition by Ab417 was the result of inhibition of tumor cell proliferation *in vivo*, Choi-CK cells were injected into nude mice to establish tumors. When the tumors had reached a size of approximately 110 mm^3^, Ab417 or PBS as a control was *i*.*p*. injected five times for 10 days into the nude mouse model (*n* = 3), then tumor sections were stained for the proliferation marker Ki-67. Ab417 treatment resulted in 30% tumor growth inhibition compared to the control, based on mean tumor volume (data not shown), and the Ab417-treated tumors showed a lower Ki-67 index compared to the control tumors, indicating that Ab417 inhibits tumor growth by inhibiting tumor cell proliferation *in vivo* (Fig 1D and S1 Fig).

### Ab417 is internalized into the cytoplasm and reduces membrane L1CAM level

**Fig 2:**
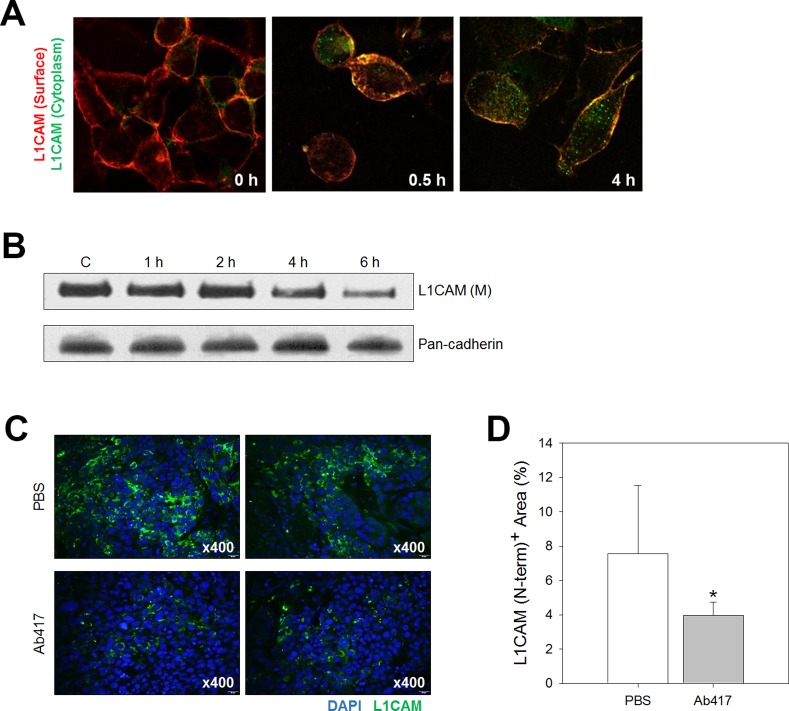
Ab417 binds to L1CAM-expressing ICC cells and reduces L1CAM levels at the membrane. (A) Confocal microscopic analysis of Ab417 internalization. SCK-L1 cells were preincubated with Ab417 (10 μg/mL) for the indicated times. Membrane L1CAM-bound Ab417 was detected using anti-Human IgG(H+L) Cross Adsorbed DyLight 594 (red), and internalized Ab417 was detected using anti-human IgG (Fc-specific)-FITC (green). (B) Choi-CK cells were incubated with Ab417 (10 μg/mL) for the indicated times, and the membrane L1CAM was detected by western blot analysis using A10-A3 and pan-cadherin as a loading control. Control (c) indicates the cells that were incubated for 6 h without Ab417. (C) Confocal microscopic images of L1CAM staining of PBS (up)- or Ab417 (down)-treated Choi-CK tumors. The images were taken at x400 magnification. L1CAM index (D) are represented as a percentage of positively-stained area comparing to total area.

Down-regulation of L1CAM expression was shown to reduce proliferation and migration of ICC cells *in vitro* and tumor growth *in vivo* [21, 24, 25]. To investigate whether inhibition of tumor cell proliferation by Ab417 was due to decreased membrane L1CAM level, we performed an antibody internalization assay using SCK-L1 cells. Internalization of Ab417 into the cytoplasm was clearly detected at 4 h after its addition to the cells (Fig 2A). Therefore, to examine whether membrane L1CAM level is reduced by internalization of Ab417, Choi-CK cells were incubated with the antibody for the indicated times (1, 2, 4, 6 h), and the membrane protein fractions of the treated cells were subjected to western blot analysis using a murine anti-L1CAM mAb (A10-A3) that binds to the Ig1 domain of human L1CAM [21]. Indeed, Ab417 down-regulated the membrane L1CAM levels in a time-dependent fashion (Fig 2B). In addition, immunofluorescence staining of the Choi-CK tumors with a murine mAb specific for the N-terminal region of human L1CAM showed that the Ab417 treated tumors had significantly reduced L1CAM level compared with the hFc treated tumors (Fig 2C and 2D).

### Biodistribution study of Ab417 in a Choi-CK xenograft model

To validate tumor targeting ability of Ab417, we performed biodistribution study of Ab417 in the Choi-CK xenograft model using ^64^Cu-NOTA-Ab417 and ^125^I-Ab417, because internalization, metabolism, and retention of radiolabeled mAbs are different based on radioisotope and radiolabeling methods: internalized ^125^I-labeled antibody is rapidly degraded in lysosomes and I-125 is excreted from the cells, whereas internalized antibody conjugated with radiometals is retained intracellularly, even after trafficking to lysosomes [30]. We prepared NOTA-Ab417 and analyzed the NOTA-to-Ab417 ratio using MALDI mass spectrometry (A and B in S2 Fig). NOTA-Ab417 was synthesized as 3.3 NOTA chelates per molecule of Ab417 antibody. The radiolabeling yield and radiochemical purity of ^125^I-Ab417 and ^64^Cu-NOTA-Ab417 were all above 99% (C and D in S2 Fig). ^64^Cu-NOTA-Ab417 showed a favorable immunoreactive index of 0.85 (S3 Fig).

**Fig 3:**
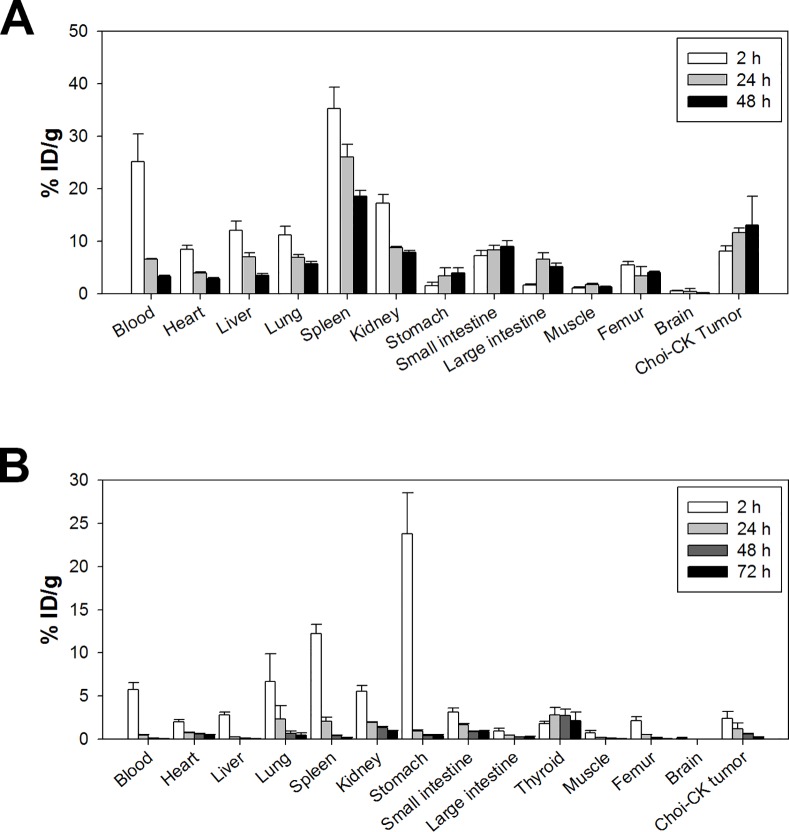
**Biodistribution study after injection of ^64^Cu-Ab417 (A) or ^125^I-Ab417 (B) in a Choi-CK xenograft model.** After tail vein injection of radio-labeled Ab417 into Choi-CK tumor xenograft mice, mice were euthanized, and radioactivity in organs was measured (*n* = 3). Radioactivity in tissues is expressed as the percentage of injected dose per gram of tissue (%ID/g).

We examined the biodistribution of ^64^Cu-NOTA-Ab417 at 2, 24, and 48 h post-injection. As shown in Fig 3A, ^64^Cu-NOTA-Ab417 was localized in Choi-CK tumors. Considering that the physical half-life of ^64^Cu is 12.7 h, the tumor uptake of ^64^Cu-NOTA-Ab417 increased and peaked at 48 h (13.1 ± 5.5%ID/g). Tumor-to-blood ratios of ^64^Cu-Ab417 increased with time: 0.3 ± 0.1 at 2 h; 1.8 ± 0.1 at 24 h; and 4.9 ± 0.4 at 48 h. Tumor-to-muscle ratios of ^64^Cu-Ab417 were 7.1 ± 0.3 at 2 h; 6.7 ± 0.9 at 24 h; 13.0 ± 2.2 at 48 h. In contrast, the uptake of ^125^I-labeled Ab417 was rapidly decreased in the tumor and other mouse tissues, which may be due to that ^125^I-labeled Ab417 was internalized into cells and rapidly degraded and deiodinated in the lysosome (Fig 3B).

The uptake of ^64^Cu-NOTA-Ab417 in mouse tissues such as spleen, kidney, and small intestine was also observed. This may be due to the facts that L1CAM is expressed in lymphoid and myelomonocytic cells, kidney tubule epithelial cells, and intestinal crypt cells and that Ab417 binds to mouse L1CAM with an affinity (K_D_, 79.16 pM), which is 2-fold higher than that for human L1CAM (K_D_, 0.24 nM) [27].

### Anti-proliferative effect of gemcitabine or cisplatin on Choi-CK cells

Given that gemcitabine and cisplatin are considered a standard regimen for advanced cholangiocarcinoma, we evaluated the effect of these drugs on the growth of Choi-CK cells by an *in vitro* proliferation assay. The results showed that gemcitabine or cisplatin decreased the proliferation of Choi-CK cells in a dose-dependent manner, with IC_50_ values of approximately 1.5 μg/mL for gemcitabine and 3.0 μg/mL for cisplatin (A in S4 Fig).

To gain insight into the mechanism by which gemcitabine or cisplatin inhibited cell growth, we examined the effect of the drugs on cell cycle machinery. Flow cytometry analysis revealed that the cell cycle was arrested at the G2/M phase after Choi-CK cells were treated with gemcitabine or cisplatin at IC_50_, while the cell cycle arrest was more increased in the cells treated with cisplatin compared to gemcitabine (B and C in S4 Fig).

### Anti-tumor efficacy of gemcitabine or cisplatin in a Choi-CK xenograft model

**Fig 4:**
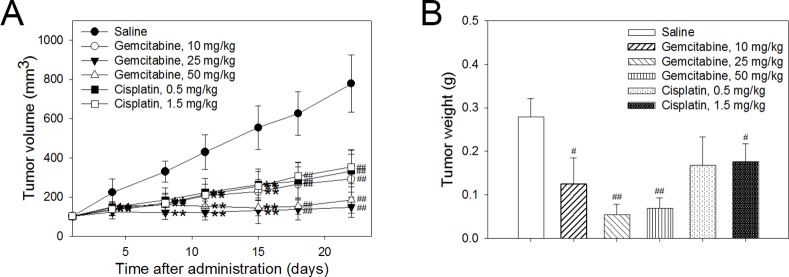
Anti-tumor efficacy of gemcitabine or cisplatin in a Choi-CK xenograft model. Drug at indicated dose was *i*.*p*. injected twice a week for 3 or 4 weeks, and tumor volume (A) and tumor weight (B) were determined. Each point indicates the mean ± s.d. *p* < 0.05 (*) and *p* < 0.01 (**), significant difference from the saline-treated group by Dunnett’s *t*-test. *p* < 0.05 (^#^) and *p* < 0.01 (^##^), significant difference from the saline group by Steel’s test.

For combined treatment with Ab417 and gemcitabine or cisplatin, we began by evaluating the anti-tumor efficacies of gemcitabine or cisplatin in the Choi-CK tumor models. Gemcitabine (10, 25, or 50 mg/kg), cisplatin (0.5 or 1.5 mg/kg), or saline (control) was intraperitoneally (*i*.*p*.) injected twice a week into nude mice bearing Choi-CK xenografts (*n* = 8), and at 22 days post-injection, tumor tissues were removed and weighed. As shown in Fig 4A and 4B, gemcitabine at 10 or 25 mg/kg inhibited tumor growth in a dose-dependent manner, with 55.4% or 80.7% tumor growth inhibition, respectively, compared to the control group, based on mean tumor weight. In contrast, cisplatin at 0.5 or 1.5 mg/kg resulted in almost the same anti-tumor efficacy, with 39.8% or 36.7% tumor growth inhibition, respectively. No treatment regimen affected body weight (A in S5 Fig).

### Anti-tumor efficacy of combined treatment with Ab417 and gemcitabine or cisplatin

**Fig 5:**
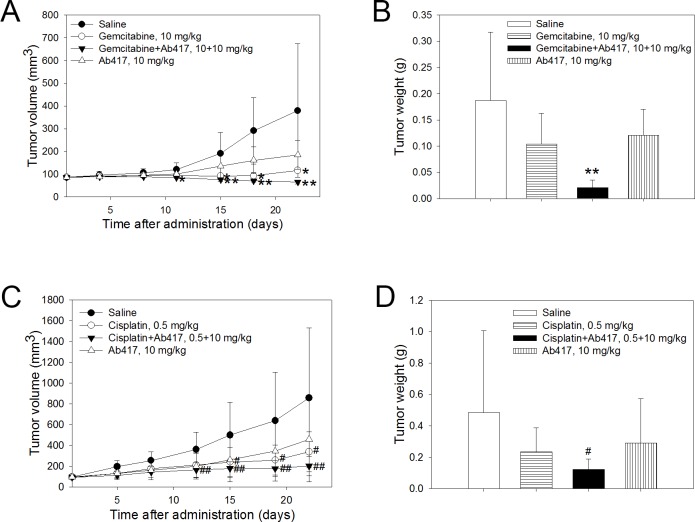
Anti-tumor efficacy of combined treatment with Ab417 and gemcitabine or cisplatin in a Choi-CK model. Tumor volume (A and C) and tumor weight (B and D) are represented. Each point indicates the mean ± s.d (*n* = 8). *p* < 0.05 (*) and *p* < 0.01 (**), significant difference from the saline group by Dunnett’s *t*-test. *p* < 0.05 (^#^) and *p* < 0.01 (^##^), significant difference from the saline group by Steel’s test.

Based on the results of previous experiments, sub-maximal doses of Ab417 and gemcitabine or cisplatin were injected into the Choi-CK xenograft model to examine whether combined treatment with Ab417 and the drug exerts greater tumor growth inhibition compared to antibody or drug alone. Ab417 (10 mg/kg), gemcitabine (10 mg/kg) or cisplatin (0.5 mg/kg), a combination of Ab417 (10 mg/kg) and gemcitabine (10 mg/kg) or cisplatin (0.5 mg/kg), or saline (control) was injected twice a week for 3 weeks into nude mice (*n* = 8) bearing Choi-CK xenografts. Combined treatment with Ab417 and gemcitabine completely inhibited tumor growth, resulting in 88.8% tumor growth inhibition compared to the control, while Ab417 or gemcitabine treatment resulted in 35.3% or 44.4% tumor growth inhibition, respectively, based on mean tumor weight (Fig 5A and 5B).

Combined treatment with Ab417 and cisplatin resulted in 79.2% (*p* < 0.01) tumor growth inhibition compared to the control, based on mean tumor weight, while treatment with Ab417 or cisplatin resulted in 39.7% or 51.2% inhibition, respectively (Fig 5C and 5D). The combined or single agent treatment did not affect body weight (B and C in S5 Fig). The results indicate that combined treatment with Ab417 and cisplatin or gemcitabine exerted enhanced tumor growth inhibition compared to treatment with antibody or drug alone.

## Discussion

We previously developed a human mAb (Ab417) having high affinities for both human and mouse L1CAM and validated its anti-tumor activity in an ICC xenograft nude mouse model [27]. In the present study, we elucidated the mode of action of Ab417 and showed that combined treatment with Ab417 and a chemotherapeutic drug for cholangiocarcinoma exerts greater tumor growth inhibition compared to antibody or drug alone. The results suggest that Ab417 in combination with chemotherapy may have potential as a new therapeutic regimen for cholangiocarcinoma. Our study is the first to show a therapeutic effect of anti-L1CAM mAb in combination with chemotherapy in cholangiocarcinoma model.

L1CAM promotes proliferation, migration, invasion, and survival of tumor cells through L1CAM homophilic interaction and/or heterophilic interactions with other cell surface molecules. Regarding mechanism of action, in this study we observed that Ab417 was internalized into L1CAM-expressing cells and decreased membrane L1CAM level *in vitro and in vivo*, while it inhibited tumor cell proliferation *in vivo*. However, it hardly showed ADCC activity *in vitro* (data not shown). In our previous study, down-regulation of cell surface L1CAM expression in ICC cells resulted in reduced tumor cell proliferation as well as AKT and FAK signaling *in vitro* and tumor growth *in vivo* [21]. Therefore, it may be likely that Ab417 exerted tumor growth inhibition *in vivo* by inducing down-regulation of L1CAM on the cell surface and thereby resulting in reduced tumor cell proliferation by a cytostatic effect.

In *in vivo* experiments, gemcitabine treatment effectively inhibited the growth of Choi-CK tumors in a dose-dependent way, but cisplatin treatment inhibited tumor growth moderately and not in a dose-dependent way, suggesting that the cells may have exerted resistance to cisplatin *in vivo*. Generally, it is known that gemcitabine causes cell cycle arrest, resulting in a cytostatic effect, whereas cisplatin, which induces DNA crosslinks, can cause cell cycle arrest and apoptosis [31]. In the present study, cell cycle was arrested at the G2/M phase after Choi-CK cells were treated with gemcitabine or cisplatin at IC_50_, while the cell cycle arrest was more increased in the cells treated with cisplatin compared to gemcitabine. Therefore, gemcitabine treatment may have inhibited tumor growth in a dose-dependent manner by a cytostatic effect. However, multiple doses of cisplatin may have induced apoptosis in the tumor cells *in vivo*, leading them to develop resistance to apoptosis and thus resulting in little dose-dependent tumor growth inhibition.

Combined treatment with Ab417 and gemcitabine or cisplatin exerted greater tumor growth inhibition compared to treatment with antibody or drug alone. The possible mechanisms may be likely that combined treatment with Ab417 and gemcitabine additively inhibited the growth of Choi-CK tumors by combined cytostatic effects, while combined treatment with Ab417 and cisplatin additively inhibited the growth of the tumors by cytostatic and cytotoxic effects. We previously observed that L1CAM conferred cisplatin resistance, while down-regulation of L1CAM expression sensitized ICC cells to cisplatin [25]. Therefore, combined treatment with Ab417 and cisplatin may have sensitized the Choi-CK cells to cisplatin, which also additively contributed to tumor growth inhibition. Whether Ab417 in combination with gemcitabine and cisplatin would improve the therapeutic response of cholangiocarcinoma compared to the combined chemotherapy remains to be established. Validation of the combination of Ab417 and chemotherapy would offer new targeted therapies with improved patient survival.

In conclusion, we clearly demonstrated the tumor targeting ability and the mode of action of Ab417 and evaluated the therapeutic efficacies of Ab417, chemotherapeutic drugs (gemcitabine or cisplatin) for cholangiocarcinoma, and combined treatment with Ab417 and a drug (gemcitabine or cisplatin) in an intrahepatic cholangiocarcinoma xenograft nude mouse model. Combined treatment with Ab417 and gemcitabine or cisplatin exerted enhanced tumor growth inhibition compared to treatment with antibody or drug alone. The results suggest that Ab417 in combination with chemotherapy may have potential as a new therapeutic regimen for cholangiocarcinoma. Our study is the first to show an enhanced therapeutic effect of a therapeutic antibody targeting L1CAM in combination with chemotherapy in cholangiocarcinoma models.

## Supporting information

S1 Materials and Methods (DOCX)

S1 Fig Immunohistochemical analysis of Choi-CK tumor xenografts. Ki-67 expression of PBS- or Ab417-treated tumor sections (*n* = 3, each row) is represented. The images were taken at x 200 magnification.
(TIF)

S2 Fig **MALDI-TOF MS of Ab417 and NOTA-Ab417 (A and B) and (C and D) ITLC radiochromatograms of** ^**125**^**I-Ab417 and** ^**64**^**Cu-NOTA-Ab417.** (A and B) The difference in mass between the molecular peaks gives a degree of conjugation of 3.3 NOTA chelates per a molecule of Ab417 antibody. a.u., arbitrary unit. (C and D) The radiolabeling yield and radiochemical purity of ^125^I-Ab417 and ^64^Cu-NOTA-Ab417 were all above 99%.
(TIF)

S3 Fig Immunoreactivity of ^64^Cu-NOTA-Ab417 in SCK-L1. Immunoreactivity test was performed and calculated by Lindmo method. Immunoreactivity of ^64^Cu-Ab417 was 0.85 (*R*^2^ = 0.97).
(TIF)

S4 Fig Cytotoxicity assay and cell cycle analysis of Choi-CK cells treated with gemcitabine or cisplatin. (A) Cells were incubated with each drug for 72 h, then, viable cell numbers were counted and relative viable cell numbers compared to drug-untreated cells were indicated. (B and C) Cells were incubated with each drug for 48 h, stained with propidium iodide, and then analyzed by flow cytometry. Percentage of cells in each phase of the cell cycle are represented.
(TIF)

S5 Fig Body weight of mice bearing Choi-CK xenografts. (A) Body weight of mice after treatment with gemcitabine or cisplatin. (B and C) Body weight of mice after combined treatment with Ab417 and gemcitabine (B) or cisplatin (C). Each point indicates the mean ± s.d.
(TIF)
