# Supplementary material for: Combination of anti-L1 cell adhesion molecule antibody and gemcitabine or cisplatin improves the therapeutic response of intrahepatic cholangiocarcinoma
Source: PLoS One. 2017 Feb 6;12(2):e0170078. doi: 10.1371/journal.pone.0170078 (PMC5293259; doi:10.1371/journal.pone.0170078)
Supplement: S1 Materials and Methods — (DOCX) [file pone.0170078.s001.docx]

**Supporting Information**

**Supporting Materials and Methods**

**MALDI-TOF mass spectroscopy**

Matrix-assisted laser desorption ionization/time of flight (MALDI/TOF) mass spectra were obtained on a ultraflextreme (Bruker Daltonics, Germany) mass spectrometer using sinapinic acid as matrix (Bruker Daltonics) in KBSI (Ohchang, Republic of Korea).

**Immunoreactivity Test**

The immunoreactivity of ^64^Cu-Ab417 was determined according to the Lindmo method [1] using lentivirally L1-CAM transduced SCK cells (SCK-L1) [2]. ^64^Cu-Ab417 (100 ng) were incubated with increasing concentration (1.25, 2.5, 5, 12.5 and 25 × 10^6^ cells/mL, *n*=3) of SCK-L1 cells. Nonspecific binding was evaluated with excess unlabeled Ab417. After 1 h incubation, the samples were washed twice in cold PBS containing 1% BSA. Each sample was counted in a gamma counter (Perkin-Elmer, WIZARD 1480). The data were plotted as a double inverse plot of the applied radiolabeled antibody over the specific binding, as a function of the inverse cell concentration. Immunoreactivity index was calculated as the inverse intercept value at the ordinate.

**References**

1. Lindmo T, Boven E, Cuttitta F, Fedorko J, Bunn PA, Jr. Determination of the immunoreactive fraction of radiolabeled monoclonal antibodies by linear extrapolation to binding at infinite antigen excess. J Immunol Methods. 1984;72: 77-89.

2. Min JK, Kim JM, Li S, Lee JW, Yoon H, Ryu CJ, et al. L1 cell adhesion molecule is a novel therapeutic target in intrahepatic cholangiocarcinoma. Clin Cancer Res. 2010;16: 3571-3580.
