# Supplementary figures and images for: Combination of anti-L1 cell adhesion molecule antibody and gemcitabine or cisplatin improves the therapeutic response of intrahepatic cholangiocarcinoma
Source: PLoS One. 2017 Feb 6;12(2):e0170078. doi: 10.1371/journal.pone.0170078 (PMC5293259; doi:10.1371/journal.pone.0170078)

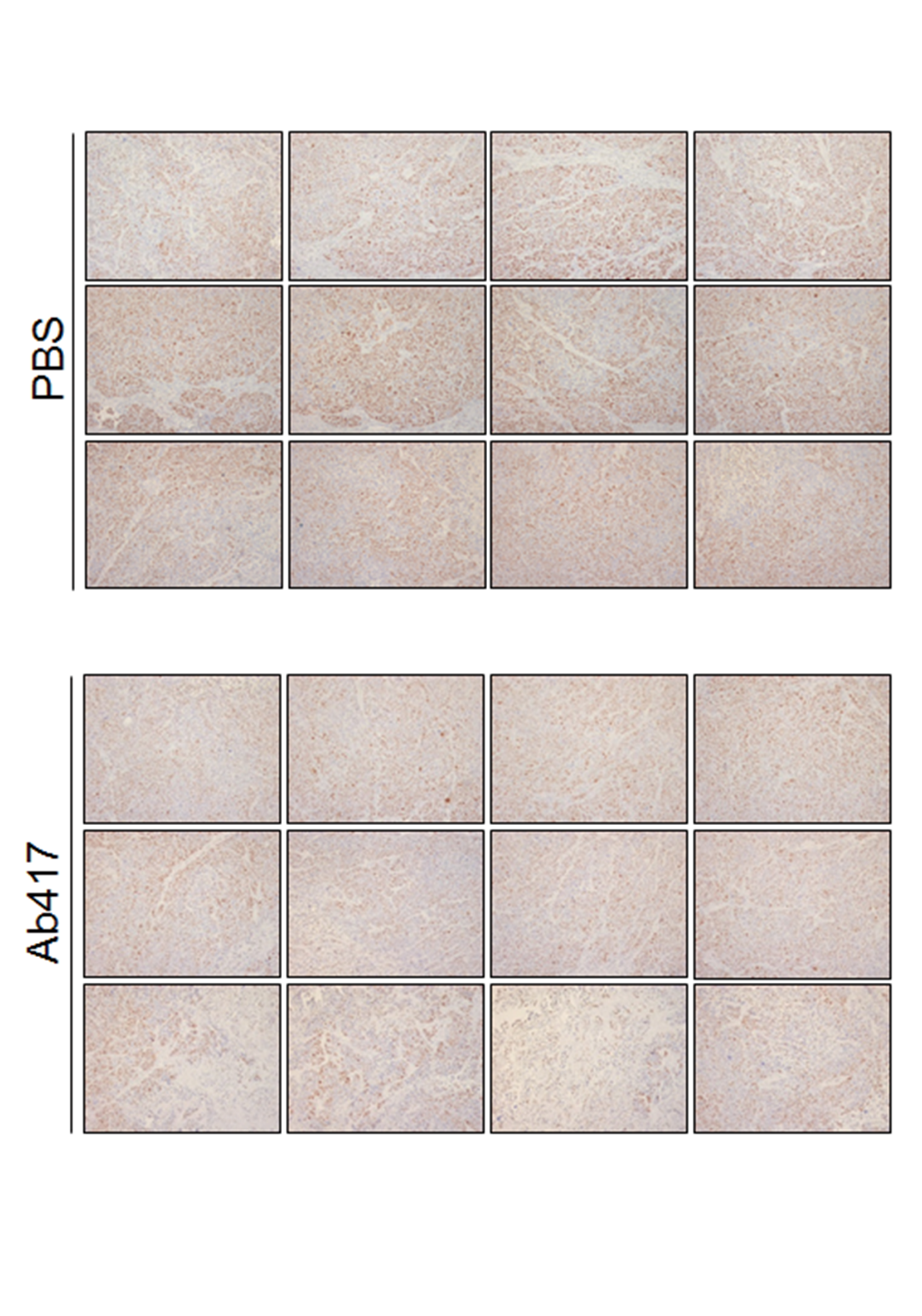

Supplement: S1 Fig — Ki-67 expression of PBS- or Ab417-treated tumor sections (n = 3, each row) is represented. The images were taken at x 200 magnification. (TIF) [file pone.0170078.s002.tif]

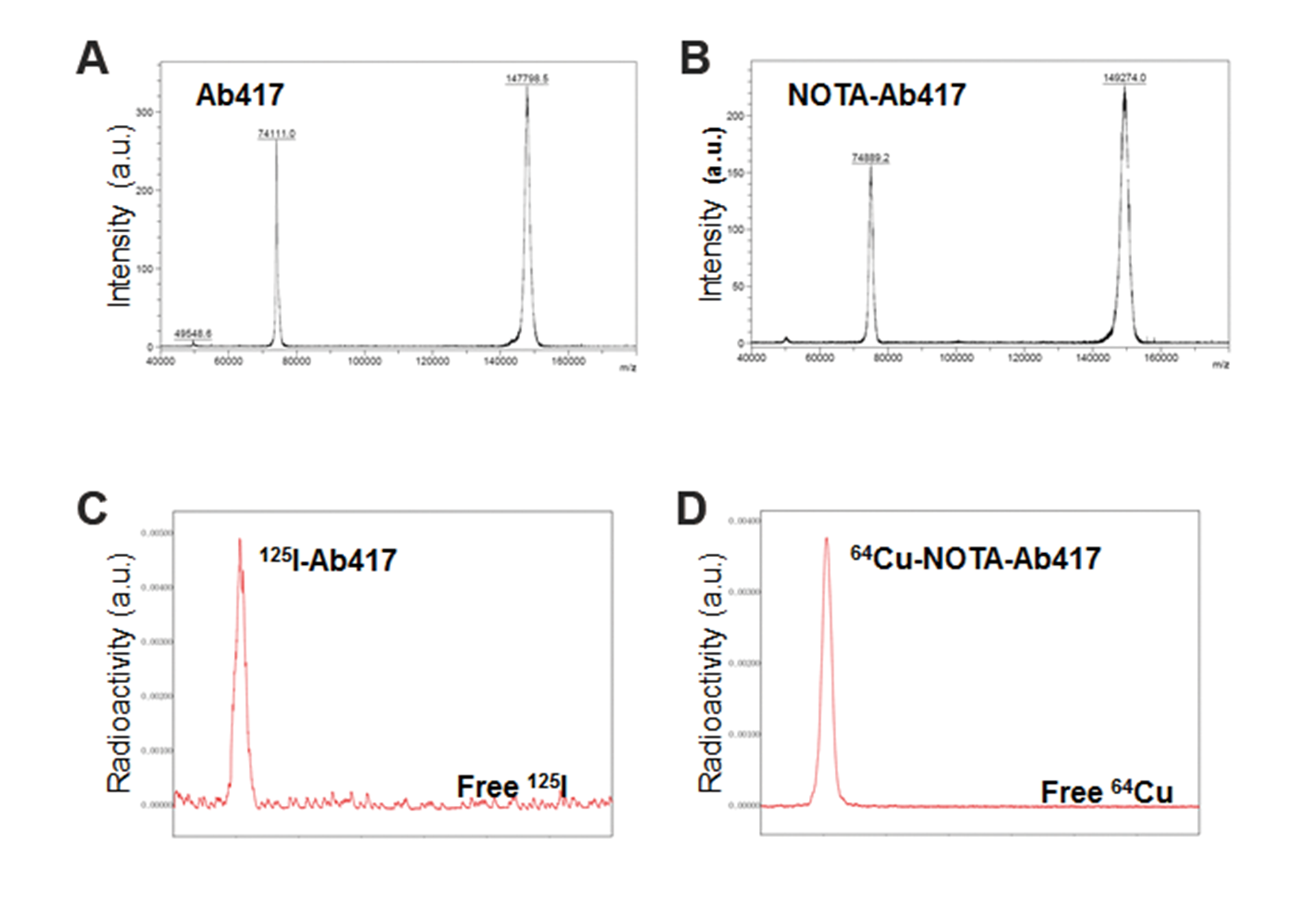

Supplement: S2 Fig — MALDI-TOF MS of Ab417 and NOTA-Ab417 (A and B) and (C and D) ITLC radiochromatograms of 125I-Ab417 and 64Cu-NOTA-Ab417. (A and B) The difference in mass between the molecular peaks gives a degree of conjugation of 3.3 NOTA chelates per a molecule of Ab417 antibody. a.u., arbitrary unit. (C and D) The radiolabeling yield and radiochemical purity of 125I-Ab417 and 64Cu-NOTA-Ab417 were all above 99%. (TIF) [file pone.0170078.s003.tif]

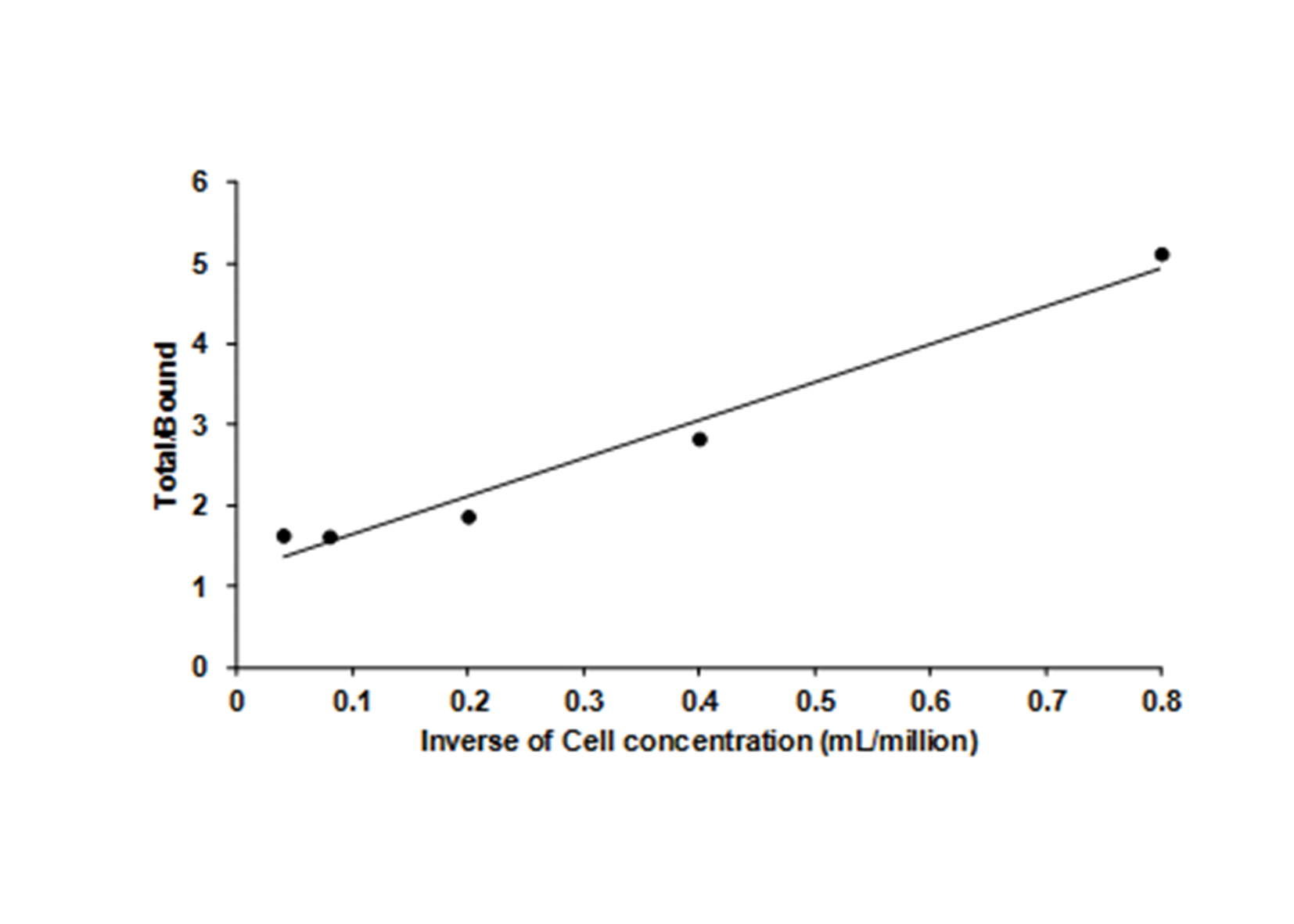

Supplement: S3 Fig — Immunoreactivity test was performed and calculated by Lindmo method. Immunoreactivity of 64Cu-Ab417 was 0.85 (R2 = 0.97). (TIF) [file pone.0170078.s004.tif]

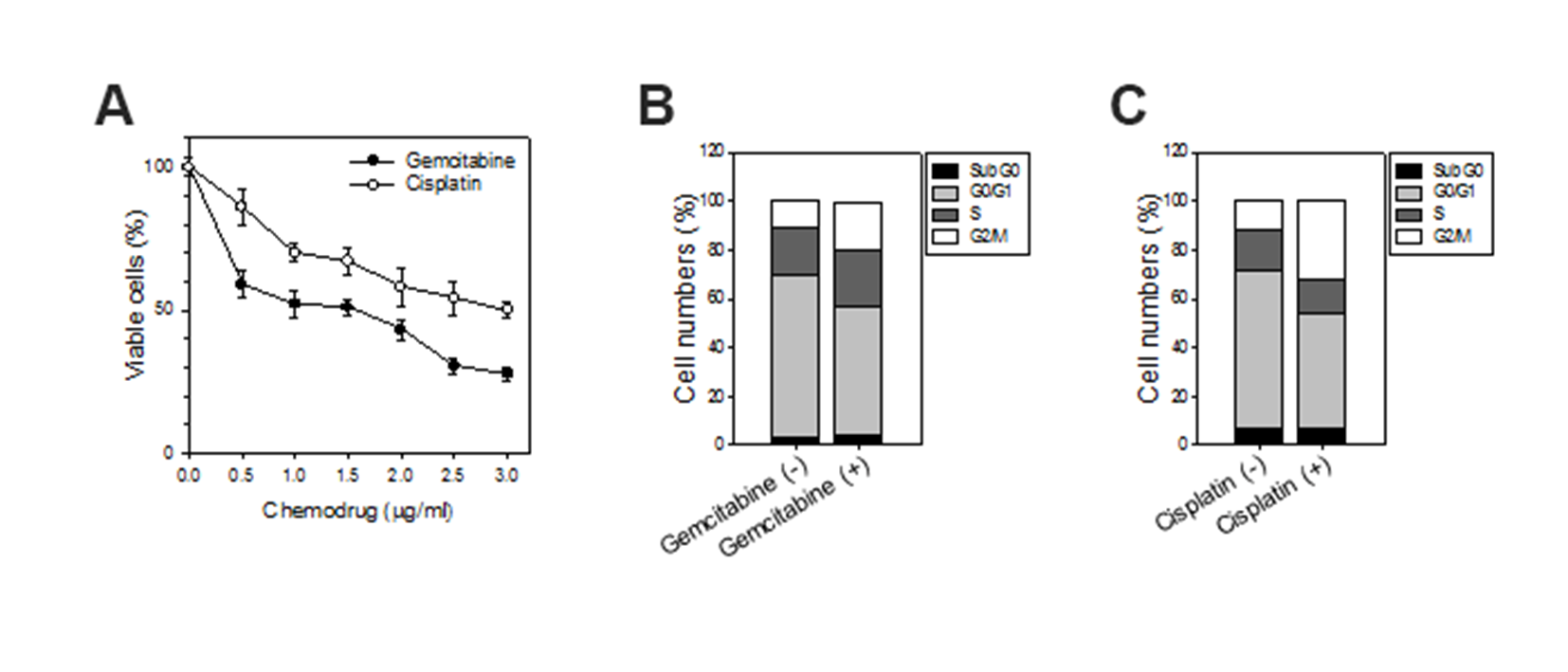

Supplement: S4 Fig — (A) Cells were incubated with each drug for 72 h, then, viable cell numbers were counted and relative viable cell numbers compared to drug-untreated cells were indicated. (B and C) Cells were incubated with each drug for 48 h, stained with propidium iodide, and then analyzed by flow cytometry. Percentage of cells in each phase of the cell cycle are represented. (TIF) [file pone.0170078.s005.tif]

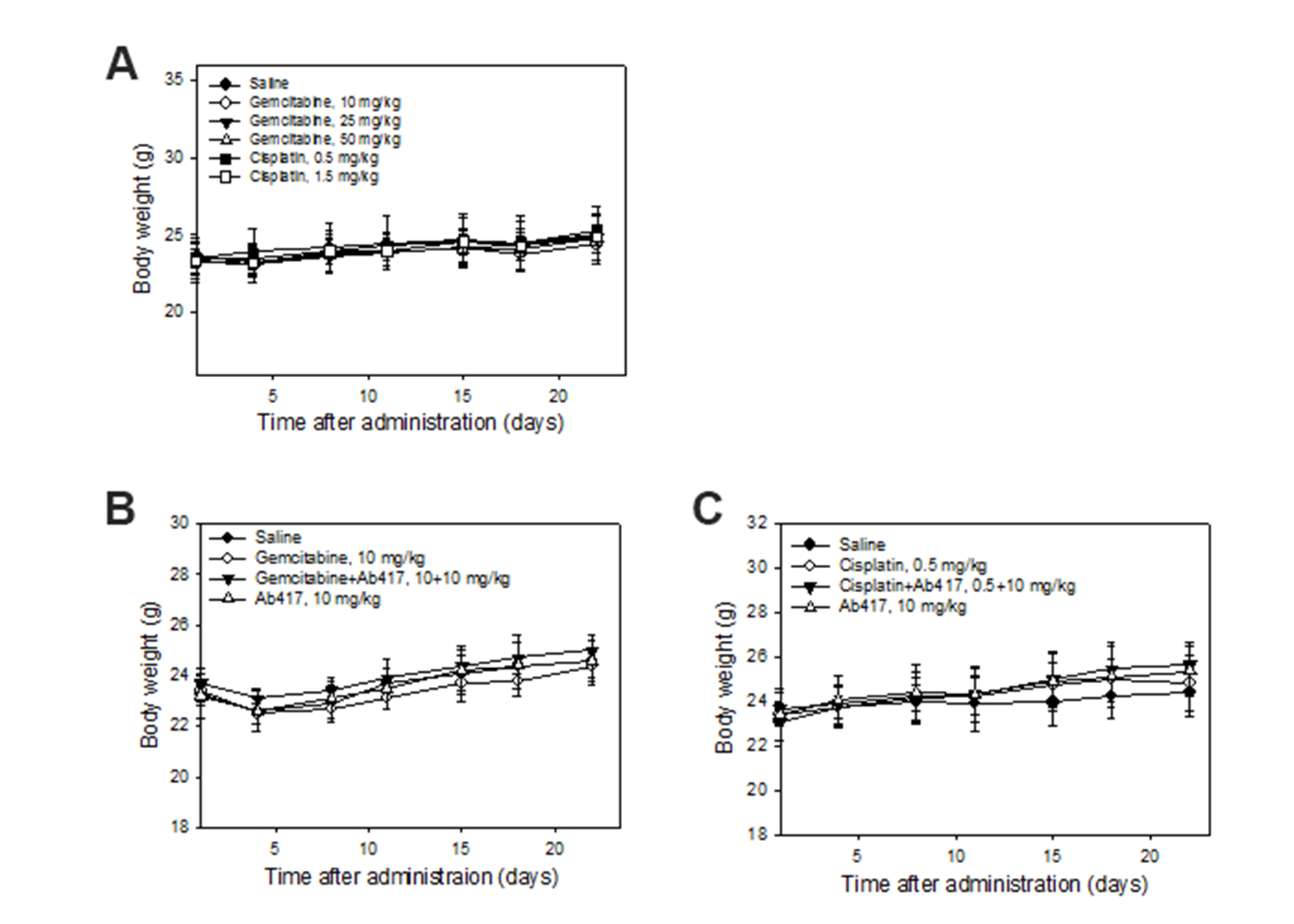

Supplement: S5 Fig — (A) Body weight of mice after treatment with gemcitabine or cisplatin. (B and C) Body weight of mice after combined treatment with Ab417 and gemcitabine (B) or cisplatin (C). Each point indicates the mean ± s.d. (TIF) [file pone.0170078.s006.tif]
